# Wernicke encephalopathy in patients with depression: A systematic review

**DOI:** 10.1111/pcn.13113

**Published:** 2020-08-06

**Authors:** Erik Oudman

**Affiliations:** ^1^ Experimental Psychology Helmholtz Institute, Utrecht University Utrecht The Netherlands; ^2^ Lelie Care Group, Korsakoff Center Slingedael Rotterdam The Netherlands

Depression is a psychiatric disorder occurring most frequently in those who have significant health problems.^1^, ^2^, ^3^, ^4^ Depression is associated with high rates of health‐care utilization and severe limitation in daily functioning.^3^, ^4^, ^5^

Poor intake of food is common in depression^6^ and nutrition can play a key role in the onset and severity of depression.^7^ In fact, a number of studies have shown an inverse association between thiamine (vitamin B1) levels and symptoms of depression in adults.^8^

A possible side‐effect of prolonged vitamin B1 deficiency is Wernicke's encephalopathy (WE), a neuropsychiatric disorder characterized by ataxia, muscle incoordination, memory loss, delirium, confusion, and ocular abnormalities. The classic triad of WE symptoms consists of ataxia, ocular abnormalities, and mental status change.^3^ Although the most common cause of WE is vitamin B1 deficiency after severe alcoholism, other causes have also been described in the literature. As descriptions in the literature have not yet been reviewed in detail, and it is relatively unknown that malnutrition in depression can lead to WE, the aim of this study was to review the clinical characteristics of WE that have developed in the context of depression in the absence of an alcohol use disorder.

The methods, flow‐chart of article selection, and references to all included case studies are presented in Appendix S1. We identified 21 case descriptions in the published literature. The average age in case descriptions was 47.2 years (SD: 16.7 years), with a range between 20 and 79 years, suggesting that both young and older patients with depression could be at risk for WE. In seven patients, diminished food intake was the primary etiology for WE in depression. In six patients, a loss of vitamins because of vomiting was the primary etiology of WE in depression. Three cases had diarrhea leading to WE, due to a loss of vitamins. Five patients had forms of cancer and a depression leading to WE, due to an increased demand for thiamine. In nine cases, weight loss was reported in detail, with an average weight loss of 14.9 kg (SD: 10.5 kg). All cases are reported in Table 1.

**Table 1:** Demographic and clinical characteristics

Author	Sex	Age (years)	Lost weight/time	Etiology	Ataxia	Eye‐movement disorder	Mental status change	MRI/CT	Thiamine treatment
Relatively uncomplicated depression									
Epstein, 1989	M	64	NA	Diarrhea and psychotic depression	+	+	+	MRI+	100 mg i.v., mildly impaired learning
Stone *et al*., 2007	M	45	NA	Water fasting diet for 44 days in severe depression	+	+, nystagmus	+	MRI+	100 mg daily, not living independently
McCormick *et al*., 2011	F	Mid‐20s	13 kg/4 months	Post‐partum depression, nausea and vomiting, borderline personality	+	+, nystagmus	+, unresponsive	MRI+	200 mg i.v., Korsakoff syndrome
Wang *et al*., 2014	F	28	NA	Major depression and motor bike collision, vomiting, extreme slimming diet	+	+	+, deteriorated consciousness	MRI+	i.v., decreased levels of consciousness
Dias *et al*., 2017	M	56	NA	Neglect of personal care in severe depression, food refusal	+	+	+ disoriented, somnolence	MRI+	500 mg i.v./day, resolved symptoms
Melchionda *et al*., 2017	M	50	NA	Loss of a job followed by depression, reduced food intake	+	–	+	MRI–	200 mg i.v./3 × per day, complete resolution
Melchionda *et al*., 2017	M	65	NA	Motor incoordination, confusion, and vomiting	+	+	+	MRI–	200 mg i.v./3 × per day, slow improvement
Odagaki *et al*., 2018	M	38	20 kg	Depression, inability to move, cachexia due to weight loss	–	–	+	MRI–	No treatment, Korsakoff syndrome
Nikolakaros *et al*., 2019	M	54	11 kg	Pain and weakness in the lower limbs, alcoholism 10 years prior to WE	+	–	+, memory loss, confabulations, disoriented	MRI+	Korsakoff syndrome
Complicated depression									
Andrade *et al*., 2010	F	27	NA	Depression and anorexia nervosa	–	–	+	MRI+	NA
Shavit & Brown, 2013	M	48	NA	Suicidal ideations and depressive symptoms, found unconscious, diabetes mellitus, osteomyelitis, hemicolectomy, and scurvy	+	+, ophthalmoplegia and nystagmus	+, loss of consciousness	NA	200 mg i.v./3 × per day, remission
Nakashima *et al*., 2013	M	43	NA	Depression with a suicide attempt, renal failure, total gastrectomy for gastric cancer	Obscured by lack of consciousness	NA	+, loss of consciousness	MRI+	500 mg/day, Korsakoff syndrome
Cocksedge & Flynn, 2014	M	68	36 kg/5 months	Lymphoma and chemotherapy, severe depression post‐diagnosis and neuroglycopenia	+	–	+, short‐term memory loss and confusion	NA	500 mg/2 × per day, complete resolution
Nikolakaros *et al*., 2016	F	42	10 kg	Depression, hypogammaglobulinemia, pyelonephritis, pneumonia, and severe urticarial	–	–	+, memory loss	MRI+	Unknown, Korsakoff syndrome
Nikolakaros *et al*., 2016	F	37	5 kg	Depression, gastroenteritis, and vomiting	+	–	+, memory and attention	MRI–	Unknown, Korsakoff syndrome
Melchionda *et al*., 2017	F	55	NA	Gastrointestinal symptoms	+	+	–	CT–	200 mg i.v./3 × per day, slow improvement
Onishi *et al*., 2018	M	79	NA	Depression and stomach cancer	–	–	+	CT–	100 mg i.v., resolution
Onishi *et al*., 2018	F	76	NA	Depression and insomnia, pancreatic cancer, insomnia	+	–	+	NA	75 mg i.v., resolution
Nikolakaros *et al*., 2018	F	33	7 kg	Total parenteral nutrition without thiamine, vomiting, diarrhea; depression, leukemia, cachexia (BMI = 16.9)	+	–	+, elevated mood, decreased sleep	MRI+	Korsakoff syndrome
Nikolakaros *et al*., 2018	M	38	26 kg	Radioactive iodine for hyperthyroidism, weight loss	+	–	+, mild confusion	MRI–	Korsakoff syndrome
Nikolakaros *et al*., 2018	M	20	6 kg	Bacterial infection, vomiting, diarrhea	+	–	+, decreased need for sleep	NA	Korsakoff syndrome

+, symptom is present; –, symptom is absent; BMI, body mass index in kg/m^2^; CT, computed tomography; F, female; M, male; MRI, magnetic resonance imaging; NA, not available.

A full WE triad was present in eight out of 21 cases. This relative occurrence of WE cases presenting with a full triad following depression seems to be higher than that seen earlier in alcoholics with WE (16%).^3^ In 20 out of 21 cases, mental status change, such as amnesia, loss of consciousness, or disorientation, was reported. In 16 out of 21 cases, ataxia was reported. Here, eight out of 21 cases were reported to show ocular signs. In 10 out of 15 case descriptions, MRI revealed radiological alterations in the thalamic area of the brain.

In 12 patients, treatment of WE was described in detail. Of importance, low levels of thiamine were given in five patients (<500 mg/day), possibly causing residual cognitive decline in three patients. Just one patient receiving higher doses of thiamine developed Korsakoff's syndrome. None of the patients received optimal thiamine dosing of three times 500 mg i.v. or i.m. per day.^9^

Depression is characterized by diminished or increased food intake.^8^ Rapidly losing weight and somatic comorbidity can lead to severe complications of depression. Patients diagnosed with depression are at risk for malnutrition. Severe malnutrition can lead to WE. Nine cases reported WE in relatively uncomplicated depression, and 12 cases reported WE in depression with somatic comorbidity.

Patients diagnosed with WE should be treated with 500 mg of thiamine i.v. or i.m./three times per day, according to recent guidelines.^3^, ^9^ Korsakoff's syndrome, a chronic neuropsychiatric disorder, developed in three out of five WE patients receiving less than 500 mg thiamine per day. Of seven WE patients who received more than 500 mg per day, only one developed Korsakoff's syndrome.

A limitation of this review is that the diagnosis of depression was not substantiated with DSM classification in the majority of reports. The nature and extent of the depression is therefore not clear in the reviewed cases.

In conclusion, depression is a risk factor for developing malnourishment. Malnourishment‐related WE is a rare but severe and preventable consequence of depression, following starvation, vomiting, or diarrhea. WE can be fully prevented by supplying prophylactic thiamine given parenterally in patients with depression. After onset of symptoms, rapid treatment with high doses of thiamine is still a life‐saving measure, directly influencing the core symptoms of WE.

## Disclosure statement

There are no conflicts of interest for the author.

## Supporting information


**Appendix S1**. Supporting information.
