# Supplementary material for: Wernicke encephalopathy in patients with depression: A systematic review
Source: Psychiatry Clin Neurosci. 2020 Aug 6;74(10):569–72. doi: 10.1111/pcn.13113 (PMC7590192; doi:10.1111/pcn.13113)
Supplement: Supplementary file 1 — Appendix S1. Supporting information. [file PCN-74-569-s001.docx]

**Supporting Information**

**Methods**

We conducted a systematic review of the literature according to the guidelines of the Preferred Reporting Items for Systematic Reviews and Meta-analysis (PRISMA) statement^1^. A comprehensive literature search was performed in MEDLINE, EMBASE, PiCarta and Scopus using “Depression” AND “Wernicke Encephalopathy” as search criteria. The last search was carried out on 28^th^ January of 2020. There were no language restrictions. All studies with a diagnosis of WE following depression were included, based on Caine’s operational criteria for WE^2^. We reviewed the title and abstract of these articles, and indexed the data for year of publication, age, sex, etiology, signs of WE, comorbid diagnosis, radiographic findings, treatment and outcome. All included studies were case reports. Cases were excluded if too little information was available to confirm a diagnosis of WE or no clinical characteristics regarding the patient or course of illness were available. Cases following alcohol use disorder were not included in the review. We included 21 cases in the review ^3-17^.

**Figure S1.** Flow chart of case study inclusion. Illustration of the number of articles identified in literature search and reasons for exclusion. Twenty-one studies met the inclusion and exclusion criteria.


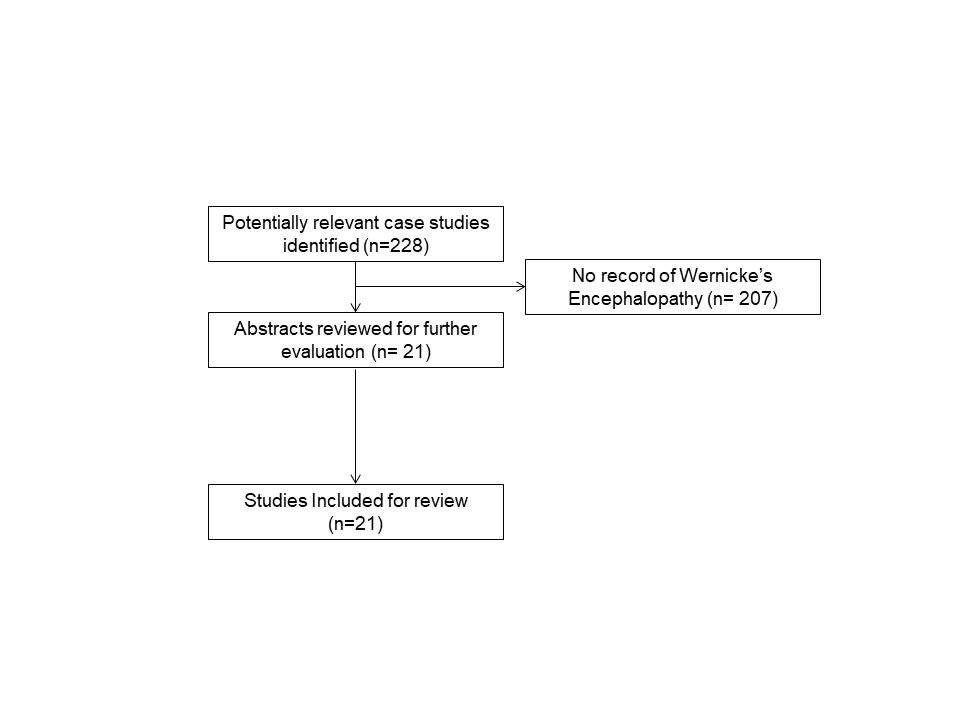


**References**
1. Moher D, Liberati A, Tetzlaff J, Altman DG, The PRISMA Group (2009). Preferred Reporting Items for Systematic Reviews and MetaAnalyses: The PRISMA Statement. PLoS Med 6(7): e1000097.

2. Caine D, Halliday GM, Kril JJ, Harper CG. Operational criteria for the classification of chronic alcoholics: identification of Wernicke's encephalopathy. JNNP 1997;62:51–60.

3.      Epstein RS. Wernicke's encephalopathy following lithium-induced
diarrhea. Am J Psychiatry. 1989;146:806-7.
4.      Stone R, Archer JS, Kiernan M. Wernicke's encephalopathy mimicking
variant Creutzfeldt-Jakob disease. J Clin Neurosci. 2008;15:1308-10.
5.      Santos Andrade C, Tavares Lucato L, da Graça Morais Martin M,
Joaquina Marques-Dias M, Antonio Pezzi Portela L, Scarabôtolo Gattás G, da Costa Leite C. Non-alcoholic Wernicke's encephalopathy: broadening the clinicoradiological spectrum. Br J Radiol. 2010;83(989):437-46.
6.      McCormick LM, Buchanan JR, Onwuameze OE, Pierson RK, Paradiso S.
Beyond alcoholism: Wernicke-Korsakoff syndrome in patients with psychiatric disorders. Cogn Behav Neurol. 2011;24:209-16.
7.      Shavit I, Brown TM. Simultaneous scurvy and Wernicke's encephalopathy
in a patient with an ascorbate-responsive dyskinesia. Psychosomatics.
2013;54:181-6.
8.      Nakashima Y, Ito K, Nakashima H, Shirakawa A, Abe Y, Ogahara S,
Sasatomi Y, Yasunaga T, Ifuku M, Tsugawa J, Tsuboi Y, Saito T. Wernicke'
s encephalopathy that developed during the introduction period of peritoneal dialysis. Intern Med. 2013;52(18):2093-7.
9.      Cocksedge KA, Flynn A. Wernicke-Korsakoff syndrome in a patient with
self-neglect associated with severe depression. JRSM Open. 2014;5(2):
2042533313518915.
10.      Wang AY, Chang WT, Chen YF, Hsieh MJ. Conscious disturbance after
collision injury in a patient on diet: Wernicke's encephalopathy. J Formos Med Assoc. 2014;113(6):392-3.
11.      Nikolakaros G, Ilonen T, Kurki T, Paju J, Papageorgiou SG, Vataja R.
Non-alcoholic Korsakoff syndrome in psychiatric patients with a history of undiagnosed Wernicke's encephalopathy. J Neurol Sci. 2016;370:296-302.
12.     Dias SP, Diogo MC, Capela C, Marques R, Gonçalves M. Wernicke's
encephalopathy due to food refusal in a patient with severe depressive disorder. J Neurol Sci. 2017;375:92-93.
13.     Melchionda D, Martino T, Carapelle E, Lalla A, Cologno D, Avolio C.
Wernicke's encephalopathy following reduced food intake due to depressive disorders. Nutr Neurosci. 2018;21(5):373-376.
14.     Odagaki Y. A Case of Non-Alcoholic Korsakoff Syndrome Resulting from
Malnutrition due to Self-Neglect and Severe Depression. Neuropsychiatry 2018;8:739-744.
15.     Onishi H, Ishida M, Tanahashi I, Takahashi T, Ikebuchi K, Taji Y,
Kato H, Akechi T. Early detection and successful treatment of Wernicke's encephalopathy in outpatients without the complete classic triad of symptoms who attended a psycho-oncology clinic. Palliat Support Care. 2018;16(5):633-636.
16.     Nikolakaros G, Kurki T, Paju J, Papageorgiou SG, Vataja R, Ilonen T.
Korsakoff Syndrome in Non-alcoholic Psychiatric Patients. Variable Cognitive Presentation and Impaired Frontotemporal Connectivity. Front Psychiatry. 2018;9:204.
17.     Nikolakaros G, Kurki T, Myllymäki A, Ilonen T. A patient with
Korsakoff syndrome of psychiatric and alcoholic etiology presenting as
DSM-5 mild neurocognitive disorder. Neuropsychiatr Dis Treat. 2019;15:
1311-1320.
